# Outcomes of elderly patients with organophosphate intoxication

**DOI:** 10.1038/s41598-021-91230-2

**Published:** 2021-06-02

**Authors:** Jia-Ruei Yu, Yi-Chou Hou, Jen-Fen Fu, I-Kuan Wang, Ming‐Jen Chan, Chao-Yu Chen, Cheng-Hao Weng, Wen-Hung Huang, Huang-Yu Yang, Ching-Wei Hsu, Tzung-Hai Yen

**Affiliations:** 1 Department of Nephrology, Clinical Poison Center, Kidney Research Center, Center for Tissue Engineering, Chang Gung Memorial Hospital, Linkou, and Chang Gung University, 199 Tung Hwa North Road, Taipei, Taoyuan, 105 Taiwan; 2 Division of Nephrology, Department of Internal Medicine, Cardinal Tien Hospital, and School of Medicine, Fu-Jen Catholic University, New Taipei City, Taiwan; 3 Department of Medical Research, Chang Gung Memorial Hospital, Linkou, and Graduate Institute of Clinical Medical Sciences, Chang Gung University, Taoyuan, Taiwan; 4 Department of Nephrology, China Medical University Hospital, China Medical University, Taichung, Taiwan

**Keywords:** Geriatrics, Outcomes research, Respiratory tract diseases

## Abstract

This study analysed the clinical patterns and outcomes of elderly patients with organophosphate intoxication. A total of 71 elderly patients with organophosphate poisoning were seen between 2008 and 2017. Patients were stratified into two subgroups: survivors (n = 57) or nonsurvivors (n = 14). Chlorpyrifos accounted for 33.8% of the cases, followed by methamidophos (12.7%) and mevinphos (11.3%). Mood, adjustment and psychotic disorder were noted in 39.4%, 33.8% and 2.8% of patients, respectively. All patients were treated with atropine and pralidoxime therapies. Acute cholinergic crisis developed in all cases (100.0%). The complications included respiratory failure (52.1%), aspiration pneumonia (50.7%), acute kidney injury (43.7%), severe consciousness disturbance (25.4%), shock (14.1%) and seizures (4.2%). Some patients also developed intermediate syndrome (15.5%) and delayed neuropathy (4.2%). The nonsurvivors suffered higher rates of hypotension (*P* < 0.001), shock (*P* < 0.001) and kidney injury (*P* = 0.001) than survivors did. Kaplan–Meier analysis indicated that patients with shock suffered lower cumulative survival than did patients without shock (log-rank test, *P* < 0.001). In a multivariate-Cox-regression model, shock was a significant predictor of mortality after intoxication (odds ratio 18.182, 95% confidence interval 2.045–166.667, *P* = 0.009). The mortality rate was 19.7%. Acute cholinergic crisis, intermediate syndrome, and delayed neuropathy developed in 100.0%, 15.5%, and 4.2% of patients, respectively.

## Introduction

Organophosphate intoxication is common in Asian populations because of easy access. In theory, the outcomes of organophosphate intoxication are split into three clear-cut clinical spectra, acute cholinergic crisis, intermediate syndrome and delayed neuropathy^1^. Acute cholinergic crisis arises rapidly following organophosphate exposure due to the acetylcholinesterase inhibition and the manifestations encompass muscarinic and nicotinic symptoms and signs^2^. Neuromuscular blockage and cerebral depression may develop and proceed to respiratory failure, consciousness disruption and mortality. Intermediate syndrome normally arises in 1–4 days, delayed neuropathy arises in 7–21 days, and both are characterized by neurologic impairment^3–6^. Intermediate syndrome is diagnosed clinically by onset of muscular paralysis involving proximal limb muscles and muscles innervated by cranial nerves, occurring in conscious patients after atropine treatment of cholinergic crisis^5^. Delayed neuropathy is a sensory-motor peripheral neuropathy and is diagnosed clinically by distal motor weakness with sparing of neck, cranial nerves, and proximal muscles, or distal sensory symptoms, such as paresthesia or numbess^6^. The intermediate syndrome presents with proximal muscle weakness and cranial nerve aberrations, while delayed polyneuropathy is exemplified by stocking and glove distribution neuropathy and sensory deficit.

Although the toxicity of organophosphate pesticides in the general population is well documented, there is still a paucity of studies that assess the outcomes of elderly patients with organophosphate intoxication. Table 1 summarizes published studies of organophosphate intoxication in elderly patients^7–14^. The mortality rates ranged between 12.9% and 22.9%. The mean age of these studies was approximately 60 years. In a retrospective study of 42 patients with Class I organophosphate intoxication, Lee et al.^7^ reported four (9.5%) patients died of pneumonia and acute respiratory distress syndrome. In another study of 26 patients with fenitrothion intoxication^8^, two (7.7%) patients died of acute lung injury. In another retrospective study^9^, 22 of 96 (22.9%) patients died. In another study of 131 patients^10^, 29 (22.1%) patients died. According to Cha et al. analysis^11^, 18 of 99 (18.2%) patients died after admission. Furthermore, a 14.6% mortality rate was observed in Moon et al. investigation^12^. In another study^13^, it was demonstrated that the in-hospital mortality rate was 19%, and nine patients died within 2 days of ingestion. Finally, in another study of 62 patients^14^, pneumonia and multiple organ failure led to mortality in 8 (12.9%) patients.

**Table 1:** Study of organophosphate intoxication in elderly patients (sample size more than 5).

Year	Study	Hospital	Area	Sample size	Time period	Types of organophosphates	Age (year)	Hospitalization length (day)	Mortality rate (%)
2011	Lee et al. ^7^	Chonnam National University Hospital	Korea	42	January 2005 to December 2010	Class I	60.5 (44.0–68.3)	18.0 (8.0–32.0)	9.5
2011	Matsuda et al. ^8^	Kawasaki Medical School Hospital	Japan	26	Not mentioned	Fenitrothion	62.4 ± 14.5	Not mentioned	7.7
2013	Lee et al. ^9^	Samsung Changwon Hospital	Korea	96	January 2007 to February 2012	Not mentioned	60.9 ± 17.1	Not mentioned	22.9
2013	Kim et al. ^10^	Samsung Changwon Hospital	Korea	131	September 2006 to December 2012	Not mentioned	61.8 ± 16.3	Not mentioned	22.1
2014	Cha et al. ^11^	Wonju Severance Christian Hospital	Korea	99	March 2008 to December 2013	Class I (15.2%), II (39.4%), III (0.0%), U (45.5%)	61.0 ± 17.0	10.0 (4.0–21.0)	18.2
2015	Moon et al. ^12^	Chonnam National University Medical School	Korea	198	2004 to 2014	Class Ia (29.3%), Ib (21.1%), II (30.8%), III (3.8%), U (3.8%)	61.0 ± 16.2	Not mentioned	14.6
2018	Lee and Lee ^13^	Chonnam National University Hospital	Korea	100	January 2011 to December 2015	Class Ia (17.0%), Ib (31.0%), II (42.0%), III (0.0%), U (10.0%)	61.0 (52.0–72.8)	14.0 (6.0–25.0)	19.0
2018	Kwon et al. ^14^	Yonsei University Wonju College of Medicine	Korea	62	March 2011 to December 2016	Dimethyl subtype: dichlorvos (n = 10), phosphamidon (n = 14), methidathion (n = 4), fenthion (n = 2). Diethyl subtype: chlorpyrifos (n = 13), parathion (n = 1), diazinon (n = 2). Others: not mentioned	60.0 ± 16.0	15.5 (8.0–29.5)	12.9
2021	Current study	Chang Gung Memorial Hospital	Taiwan	71	2008 to 2017	Class Ia (16.9%), Ib (19.7%), II (60.6%), III (2.8%), U (0.0%)	70.8 ± 7.7	20.4 ± 16.9	19.7

Age and hospitalization length are presented as the mean ± standard deviation or median with interquartile range. Pesticide toxicity was categorized as follows, according to World Health Organization recommendations: Ia extremely hazardous, Ib highly hazardous, II moderately hazardous, III slightly hazardous and U unlikely to present acute hazards^42^.

Some clinical studies^15–18^ reported that aging was a risk factor for mortality in organophosphate intoxication. For example, Liu et al.^15^ demonstrated that decreasing pH values (pH < 7.2) and increasing age (≥ 50 years) were associated with mortality in patients with organophosphate intoxication. This could be explained by poor health conditions in the elderly patients. In addition, some laboratory studies^19,20^ reported that aged animals were more sensitive to cholinergic (muscarinic) agonists. This may be associated with age-related changes in cholinergic neurochemical processes due to a reduction in muscarinic receptors with aging^21^.

According to the National Development Council of Taiwan^22^, Taiwan shall become a super-aged society in which at least 20% of the population is 65 or older by 2026. Acute pesticide intoxication is a common method of suicide globally^23^. Elderly people are a sensitive group in terms of pesticide intoxication because many farm workers are elderly people rather than young people. Young people often tend to seek work in cities. Therefore, this study attempted to analyse the clinical patterns and outcomes of mortality in elderly patients with organophosphate intoxication.

## Results

Table 2 outlines the baseline demographic data of 71 elderly patients with organophosphate poisoning, stratified according to status at discharge as survivors (n = 57) or nonsurvivors (n = 14). The patients were 70.8 ± 7.7 years old, and most patients were male (60.6%). Chlorpyrifos accounted for one-third of the cases (33.8%), followed by methamidophos (12.7%) and mevinphos (11.3%). The majority of exposures occurred via the oral route (88.7%), but some occurred via the dermal route (11.3%). Hypertension, diabetes mellitus and chronic kidney disease were found in 45.1%, 12.7% and 7.0% of patients, respectively. Many of the patients did not have a job (42.3%) or worked as farmers (32.4%). Mood, adjustment and psychotic disorder were noted in 39.4%, 33.8% and 2.8% of patients, respectively. Notably, it was revealed that the nonsurvivors had a higher rate of chronic kidney disease than survivors did (21.4% versus 3.6%, *P* = 0.015). Otherwise, there were no significant differences in baseline variables between the groups.

**Table 2:** Baseline characteristics of elderly patients with organophosphate poisoning, stratified according to status at discharge as survivors or nonsurvivors (n = 71).

Variable	All patients (n = 71)	Survivors (n = 57)	Nonsurvivors (n = 14)	*P*
Age, years	70.8 ± 7.7	70.0 ± 7.8	73.6 ± 6.3	0.115
Male, n (%)	43 (60.6)	36 (63.2)	7 (50.0)	0.380
Time of admission, hour	44.7 ± 87.19 [3.9 (0.2–336.0)]	48.8 ± 92.1 [3.9 (0.5–336.0)]	28.3 ± 66.4 [3.7 (0.2–192.0)]	0.559
Organophosphate type, n (%)				0.897
Acephate	2 (2.8)	2 (3.5)	0 (0)	
Chlopyrifos	24 (33.8)	18 (31.6)	6 (42.9)	
Diazinon	1 (1.4)	0 (0)	1 (7.1)	
Dichlorvos	1 (1.4)	1 (1.8)	0 (0)	
Dicrotophos	1 (1.4)	1 (1.8)	0 (0)	
Dimethoate	6 (8.5)	4 (7.0)	2 (14.3)	
Ethion	1 (1.4)	1 (1.8)	0 (0)	
Fenamiphos	1(1.4)	1 (1.8)	0(0)	
Malathion	2 (2.8)	2 (3.5)	0(0)	
Metasystox I	1 (1.4)	1 (1.8)	0(0)	
Methamidophos	9 (12.7)	6 (10.5)	3(21.4)	
Methidathion	1 (1.4)	1 (1.8)	0 (0)	
Mevinphos	8 (11.3)	7 (12.3)	1 (7.1)	
Parathion	3 (4.2)	3 (5.3)	0 (0)	
Phenthoate	1 (1.4)	1 (1.8)	0 (0)	
Phorate	1 (1.4)	1 (1.8)	0 (0)	
Profenofos	5 (7.0)	4 (7.0)	1 (7.1)	
Sumithion	2 (2.8)	2 (3.5)	0 (0)	
Trichlorfon	1 (1.4)	1 (1.8)	0 (0)	
Organophosphate toxicity, n (%)				0.606
Ia extremely hazardous	12 (16.9)	11 (19.3)	1 (7.1)	
Ib highly hazardous	14 (19.7)	11 (19.3)	3 (21.4)	
II moderately hazardous	43 (60.6)	33 (57.9)	10 (71.4)	
III slightly hazardous	2 (2.8)	2 (3.5)	0 (0)	
U unlikely to present acute hazard	0 (0)	0 (0)	0 (0)	
Occupation, n (%)				0.055
No occupation	37 (52.1)	26 (45.6)	11 (78.6)	
Farmer	23 (32.4)	22 (38.6)	1 (7.1)	
Non-farmer	11 (15.5)	9 (15.8)	2 (14.3)	
Marriage, n (%)				1.000
Married	55 (77.5)	44 (77.2)	11 (78.6)	
Others (unmarried, divorced, widowed)	16 (22.5)	13 (22.8)	3 (21.4)	
Route of exposure, n (%)				0.342
Oral	63 (88.7)	49 (86.0)	14 (100)	
Dermal	8 (11.3)	8 (14.0)	0 (0)	
Intentional, n (%)	63 (88.7)	49 (86.0)	14 (100.0)	0.342
**Comorbidities**				
Hypertension, n (%)	32 (45.1)	25 (43.9)	7 (50.0)	0.768
Diabetes mellitus, n (%)	9 (12.7)	8 (14.0)	1 (7.1)	0.677
Chronic kidney disease, n (%)				0.015*
None	66 (93.0)	55 (96.4)	11 (78.6)	
Stage 1	0 (0)	0 (0)	0 (0)	
Stage 2	0 (0)	0 (0)	0 (0)	
Stage 3	4 (5.6)	1 (1.8)	3 (21.4)	
Stage 4	0 (0)	0 (0)	0 (0)	
Stage 5	1 (1.4)	1 (1.8)	0 (0)	
Chronic obstructive pulmonary disease, n (%)	2 (2.8)	1 (1.8)	1 (7.1)	0.358
Malignancy, n (%)	5 (7.0)	5 (8.8)	0 (0)	0.575
Mood disorder, n (%)	28 (39.4)	22 (38.6)	6 (42.9)	0.770
Adjustment disorder, n (%)	24 (33.8)	17 (29.8)	7 (50.0)	0.209
Psychotic disorder, n (%)	2 (2.8)	1 (1.8)	1 (7.1)	0.358
Smoking habit, n (%)	20 (28.2)	18 (31.6)	2 (14.3)	0.321
Alcohol consumption, n (%)	21 (29.6)	19 (33.3)	2 (14.3)	0.205

Categorical variables were presented as numbers with percentages in brackets, while continuous variables were presented as the means and standard deviations. Time of admission was presented as means and standard deviations as well as median and range. *P < 0.05.

As shown in Table 3, organophosphate intoxication was associated with acute cholinergic crisis and often resulted in severe medical complications, including respiratory failure (52.1%), aspiration pneumonia (50.7%), acute kidney injury (43.7%), severe consciousness disturbance (25.4%), shock (14.1%), and seizures (4.2%). Out of the 71 patients, 11 (15.5%) patients developed intermediate syndrome and three (4.2%) patients developed delayed neuropathy. Two patients with intermediate syndrome developed delayed neuropathy. One patient with intermediate syndrome died of shock in 16 days. The case was an 88 year-old female with a past history of old cerebral infarct who committed suicide by ingestion of diazinon pesticide. She was admitted to our hospital in 0.2 h, and the acute cholinergic syndrome was managed intensively. Intermediate syndrome developed in 50 h after exposure. Furthermore, she was complicated with aspiration pneumonia, acute kidney injury and acute respiratory failure during hospitalization. In addition, the nonsurvivors suffered higher incidence rates of hypotension (100.0% versus 24.6%, *P* < 0.001), shock (64.3% versus 1.8%, *P* < 0.001) and acute kidney injury (85.7% versus 33.3%, *P* = 0.001) than survivors did.

**Table 3:** Clinical findings of elderly patients with organophosphate poisoning, stratified according to status at discharge as survivors or nonsurvivors (n = 71).

Variable	All patients (n = 71)	Survivors (n = 57)	Nonsurvivors (n = 14)	*P*
1. Acute cholinergic crisis				
**Respiratory system**				
Shortness of breath, n (%)	34 (47.9)	26 (45.6)	8 (57.1)	0.554
Respiratory failure, n (%)	37 (52.1)	27 (47.4)	10 (71.4)	0.140
Aspiration pneumonia, n (%)	36 (50.7)	29 (50.9)	7 (50.0)	1.000
**Cardiovascular system**				
Systolic blood pressure, mmHg	150.9 ± 33.6	154.0 ± 27.9	137.6 ± 51.2	0.146
Diastolic blood pressure, mmHg	82.7 ± 21.1	83.9 ± 15.1	77.6 ± 38.1	0.372
Heart rate, bpm	91 ± 22	92 ± 21	90 ± 24	0.851
Corrected QT-interval prolongation, n (%)	15 (21.1)	11 (19.3)	4 (28.6)	0.475
Hypotension, n (%)	28 (39.4)	14 (24.6)	14 (100)	< 0.001***
Shock, n (%)	10 (14.1)	1 (1.8)	9 (64.3)	< 0.001***
**Gastrointestinal system**				
Emesis, n (%)	26 (36.6)	23 (40.4)	3 (21.4)	0.230
Diarrhea, n (%)	21 (29.6)	17 (29.8)	4 (28.6)	1.000
**Central nervous system**				
Glasgow Coma scale, n (%)				0.231
Mild injury	44 (62.0)	37 (64.9)	7 (50.0)	
Moderate injury	9 (12.7)	8 (14.0)	1 (7.1)	
Severe injury	18 (25.4)	12 (21.1)	6 (42.9)	
Seizure, n (%)	3 (4.2)	2 (3.5)	1 (7.1)	0.488
Delirium, n (%)	13 (18.3)	13 (22.8)	0 (0)	0.058
**Genitourinary system**				
Acute kidney injury, n (%)	31 (43.7)	19 (33.3)	12 (85.7)	0.001**
2. Intermediate syndrome, n (%)	11 (15.5)	10 (17.5)	1 (7.1)	0.680
3. Delayed neuropathy, n (%)	3 (4.2)	3 (5.3)	0 (0)	1.000
Time of death after exposure, day			6.8 ± 6.7 [4.0 (1.0–20.2)]	

Categorical variables were presented as numbers with percentages in brackets, while continuous variables were presented as the means and standard deviations. Time of death after exposure was presented as means and standard deviations as well as median and range. **P < 0.01, ***P < 0.001.

Table 4 revealed that the nonsurvivors suffered lower blood cholinesterase levels (initial 0.53 ± 0.30 U/mL versus 3.20 ± 3.84 U/mL, *P* = 0.012; nadir 0.43 ± 0.30 U/mL versus 2.56 ± 3.18 U/mL, *P* = 0.025) than survivors did. Moreover, the nonsurvivors suffered higher blood levels of creatinine (initial 1.75 ± 1.21 mg/dL versus 1.09 ± 0.90 mg/dL, *P* = 0.025; nadir 3.40 ± 2.15 mg/dL versus 1.41 ± 1.32 mg/dL, *P* = 0.001) and alanine aminotransferase (initial 46.09 ± 55.21 U/L versus 27.12 ± 15.23 U/L, *P* = 0.035; nadir 144.50 ± 225.76 U/L versus 57.38 ± 47.91 U/L, *P* = 0.047) than survivors did. Finally, the nonsurvivors demonstrated poorer arterial blood gas measurements than survivors did.

**Table 4:** Laboratory data of elderly patients with organophosphate poisoning (n = 71), stratified according to mortality status.

Variable	All patients (n = 71)	Survivors (n = 57)	Nonsurvivors (n = 14)	*P*
Hemoglobin, g/dL [normal: male 13.5–17.5, female 12.0–16.0]	14.0 ± 2.6	13.1 ± 1.7	14.6 ± 4.8	0.284
Blood urea nitrogen, mg/dL [normal: 7.0–25.0]	20.7 ± 16.8	19.4 ± 17.5	25.0 ± 13.6	0.313
Creatinine (initial), mg/dL [normal: female 0.44–1.03, male 0.64–1.27, 0–18 years 0.20–1.00]	1.22 ± 1.00	1.09 ± 0.90	1.75 ± 1.21	0.025*
Creatinine (peak), mg/dL [normal: female 0.44–1.03, male 0.64–1.27, infant-18 years 0.20–1.00]	1.78 ± 1.68	1.41 ± 1.32	3.40 ± 2.15	0.001**
Alanine aminotransferase (initial), U/L [normal: adult ≤ 36.0, < 1 year 13.0–45.0, 1–18 years 7.0–40.0]	30.5 ± 27.3	27.1 ± 15.2	46.1 ± 55.2	0.035*
Alanine aminotransferase (peak), U/L [normal: adult ≤ 36.0, < 1 year 13.0–45.0, 1–18 years 7.0–40.0]	71.1 ± 99.2	57.4 ± 47.9	144.5 ± 225.8	0.047*
Sodium, mEq/L [normal: 136.0–146.0]	140.5 ± 3.9	140.5 ± 3.2	140.3 ± 6.2	0.844
Potassium, mEq/L [normal: 3.5–5]	3.6 ± 0.5	3.6 ± 0.4	3.8 ± 0.8	0.326
Calcium, mg/dL [normal: 8.6–10.3]	8.4 ± 1.4	8.4 ± 0.6	8.5 ± 3.1	0.852
Cholinesterase (initial), U/mL [normal: 7.0–19.0]	2.7 ± 3.6	3.2 ± 3.8	0.5 ± 0.3	0.012*
Cholinesterase (nadir), U/mL [normal: 7.0–19.0]	2.2 ± 3.0	2.6 ± 3.2	0.4 ± 0.3	0.025*
C-reactive protein, mg/dL [normal < 5.0]	58.8 ± 55.59	61.1 ± 58.3	40.3 ± 21.5	0.488
Amylase, U/L [normal: 28.0–100.0]	249.8 ± 224.17	206.9 ± 177.4	361.4 ± 312.5	0.199
Lipase, U/L [normal: 11.0–82.0]	106.2 ± 87.3	79.8 ± 50.6	176.7 ± 137.4	0.102
Creatine kinase-MB, ng/mL [normal: 0.6–6.3]	14.7 ± 28.3	13.5 ± 30.9	17.3 ± 23.4	0.790
Troponin-I, ng/mL [normal: < 0.3]	0.4 ± 1.1	0.4 ± 1.1	0.6 ± 1.0	0.676
**Arterial blood gas (initial)**				
pH [normal: male 7.34–7.44, female 7.35–7.45]	7.36 ± 0.13	7.37 ± 0.12	7.29 ± 0.16	0.079
pCO2, mmHg [normal: male 35.0–45.0, female 32.0–42.0]	39.2 ± 14.3	38.0 ± 11.8	42.9 ± 21.5	0.298
pO2, mmHg [normal: 75.0–100.0]	129.5 ± 88.8	139.7 ± 94.1	92.0 ± 53.5	0.099
Bicarbonate, mm/L [normal: male 22.0–26.0, female 20.0–24.0]	21.1 ± 6.3	21.4 ± 5.3	20.3 ± 9.5	0.628
Base excess, mm/L [normal: male 2.3–2.4, female 1.2–3.3]	− 4.4 ± 7.2	− 3.9 ± 6.1	− 6.4 ± 10.3	0.289
Saturation, % [normal: 95.0–98.0]	92.3 ± 13.3	93.8 ± 10.2	87.1 ± 20.8	0.124
**Arterial blood gas (worst)**				
pH [normal: male 7.34–7.44, female 7.35–7.45]	7.21 ± 0.16	7.24 ± 0.16	7.12 ± 0.16	0.035*
pCO2, mmHg [normal: male 35.0–45.0, female 32.0–42.0]	56.7 ± 27.4	60.0 ± 30.0	48.0 ± 17.3	0.221
pO2, mmHg [normal: 75.0–100.0]	126.8 ± 99.0	142.9 ± 109.1	84.4 ± 46.0	0.095
Bicarbonate, mm/L [normal: male 22.0–26.0, female 20.0–24.0]	22.0 ± 7.5	24.3 ± 6.52	16.0 ± 6.6	0.001**
Base excess, mm/L [normal: male 2.3–2.4, female 1.2–3.3]	− 5.2 ± 8.6	− 3.0 ± 7.3	− 12.3 ± 8.8	0.003**
Saturation, % [normal: 95.0–98.0]	89.7 ± 14.6	92.6 ± 10.1	82.2 ± 21.5	0.044*

Continuous variables were presented as the means and standard deviations. *P < 0.05, **P < 0.01; pCO2 partial pressure of carbon dioxide; pO2; partial pressure of oxygen.

All (100.0%) patients were treated with atropine and pralidoxime therapies (Table 5). Three (4.2%) patients with seizure were given anticonvulsive drug. Fourteen patients (19.7%) died despite intensive treatment. Furthermore, the length of hospitalization was shorter in nonsurvivors than in survivors (6.1 ± 6.2 versus 23.9 ± 16.9 days, *P* < 0.001).

**Table 5:** Outcomes of elderly patients with organophosphate poisoning, stratified according to status at discharge as survivors or nonsurvivors (n = 71).

Variable	All patients (n = 71)	Survivors (n = 57)	Nonsurvivors (n = 14)	*P*
Gastric lavage, n (%)	47 (66.2)	37 (64.9)	10 (74.7)	0.759
Active charcoal, n (%)	31 (43.7)	24 (42.1)	7 (50.0)	0.765
Atropine, n (%)	71 (100.0)	57 (100.0)	14 (100.0)	1.000
Administrated atropine, mg	65.3 ± 101.3	72.0 ± 107.7	31.9 ± 518	0.257
Pralidoxime, n (%)	71 (100.0)	57 (100.0)	14 (100.0)	1.000
Administrated pralidoxime, g	123.9 ± 142.5	132.9 ± 151.1	78.7 ± 77.9	0.276
Anticonvulsive drug, n (%)	3 (4.2)	2 (3.5)	1 (7.1)	0.488
Hospitalization length, day	20.4 ± 16.9	23.9 ± 16.9	6.1 ± 6.2	< 0.001***

Categorical variables were presented as numbers with percentages in brackets, while continuous variables were presented as the means and standard deviations. ***P < 0.001.

**Figure 1:**
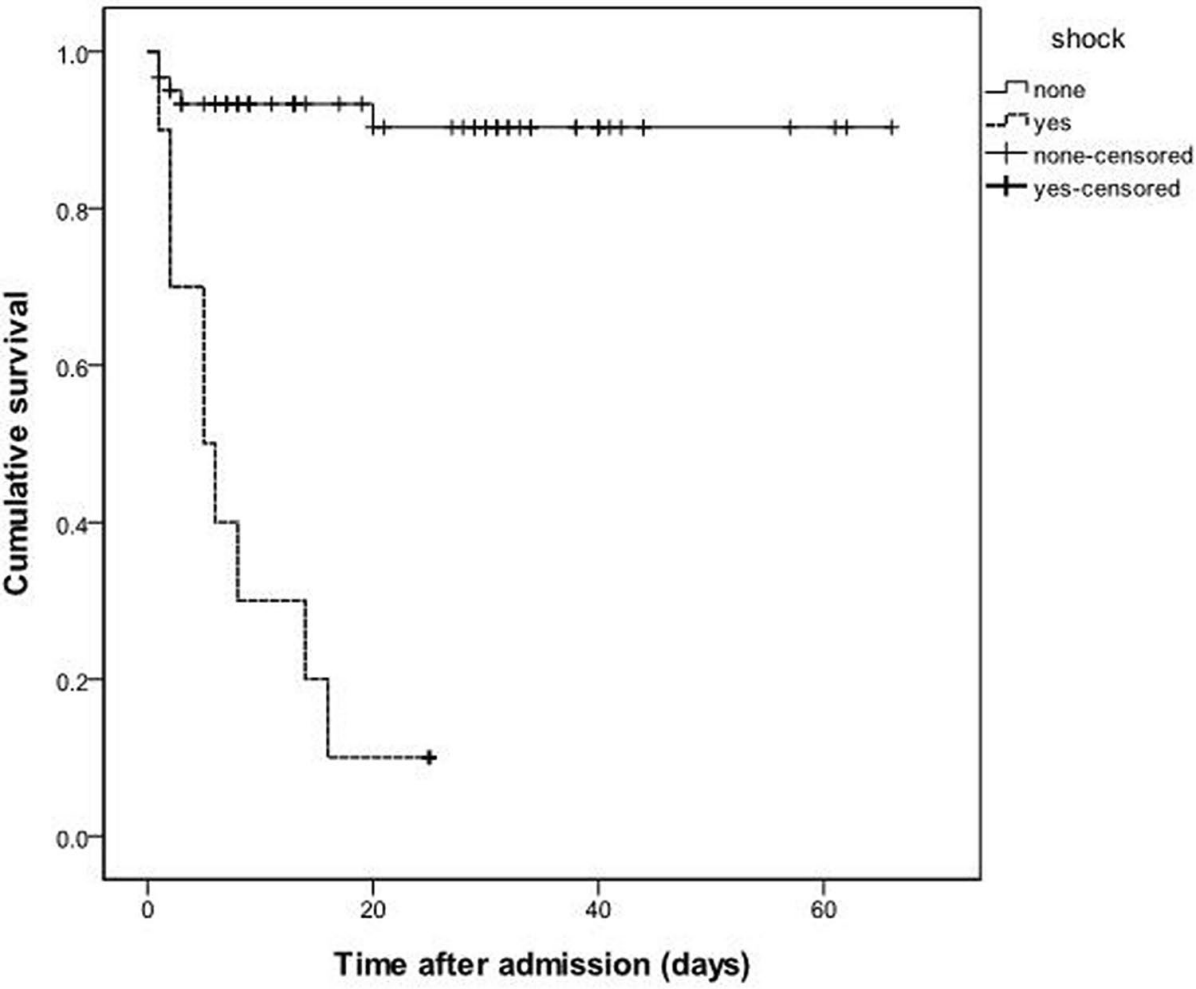
Kaplan–Meier analysis. Patients with shock suffered lower cumulative survival than patients without shock (log-rank test, chi-square = 42.704, *P* < 0.001).

In a multivariate Cox regression model (Table 6), shock was a significant predictor of mortality after organophosphate intoxication (odds ratio 18.182, 95% confidence interval 2.045–166.667, *P* = 0.009). Kaplan–Meier analysis indicated that patients with shock suffered lower cumulative survival than did patients without shock (Fig. 1, log-rank test, chi-square = 42.704, *P* < 0.001).

**Table 6:** Cox regression analysis for mortality (n = 71).

Variable	Univariate analysis			Multivariate analysis		
	Odds ratio	95% confidence interval	*P*	Odds ratio	95% confidence interval	*P*
Shock (yes)	16.129	5.263–50.000	< 0.001***	18.182	2.045–166.667	0.009**
Creatinine (per 1 mg/dL increase)	1.383	1.105–1.732	0.005**	1.335	0.896–1.988	0.155
Alanine aminotransferase (per 1 U/L increase)	1.016	1.002–1.029	0.021*	1.034	0.986–1.085	0.169
Bicarbonate (per 1 mm/L increase)	0.877	0.805–0.956	0.003**	0.989	0.884–1.107	0.849

*P < 0.05; **P < 0.01; ***P < 0.001.

## Discussion

Literature on the mortality data of elderly patients with organophosphate intoxication has been limited. The overall mortality rate was 19.7% in this study. Acute cholinergic crisis, intermediate syndrome, and delayed neuropathy developed in 100.0%, 15.5%, and 4.2% of patients, respectively.

A substantial proportion of the elderly organophosphate patients (52.1%) suffered respiratory failure necessitating mechanical ventilatory support. In a review article, Giyanwani et al.^24^ found that 491 of 1996 (24.6%) patients suffered acute respiratory failure after organophosphate intoxication. One of the overwhelming cholinergic features of organophosphate intoxication is respiratory failure. The mechanisms of respiratory failure following organophosphate intoxication involve three components: respiratory center depression, respiratory muscle weakness, or cholinergic pulmonary effect (bronchospasm and bronchorrhea)^24^. Relatively impaired baseline functions of these systems in elderly adults may worsen respiratory depression during pesticide intoxication.

Notably, 15.5% of the elderly organophosphate patients suffered intermediate syndrome. Published incidence rates of intermediate syndrome vary between 17 and 80%^25^. Intermediate syndrome is another cause of respiratory failure, as it predominantly leads to proximal muscle weakness. There are limited published data on the predictors of intermediate syndrome. In a study, Indira et al.^3^ found that age ≥ 45, Poisoning Severity Score > 2 upon admission, and Glasgow Coma Scale score ≤ 10 were associated with a risk of developing intermediate syndrome. For other factors, biochemical parameters such as serum cholinesterase, blood glucose, and oxidative stress markers have been reported to be significantly different between patients with and without intermediate syndrome^26,27^. Nevertheless, neurophysiological testing remains the gold standard for the diagnosis of intermediate syndrome.

Although not statistically significant, the time of admission for non-survivors was shorter than for survivors (48.8 ± 92.1 versus 28.3 ± 66.4 h, *P* = 0.559, Table 2), implying that the ingested dose was much higher and caused more serious symptoms sooner. Even though it was difficult to state the organophosphate dose patients were exposed to, the measured nadir cholinesterase activity could be a certain indicator of the dose, which was obviously much lower in non-survivors (2.56 ± 3.18 versus 0.43 ± 0.30 U/mL, *P* = 0.025, Table 4).

Two patients with intermediate syndrome developed delayed neuropathy. Although the pathophysiologic mechanism of intermediate syndrome is still unclear, organophosphate induced delayed neuropathy can be explained by inhibition of neuropathy-target esterase. Literature on the relationship between intermediate syndrome and delayed neuropathy after organophosphate poisoning is lacking, although case of organophosphates poisoning complicated by acute cholinergic crisis, intermediate syndrome and delayed neuropathy has been reported in literature^28^.

Shock was a significant predictor of mortality after organophosphate intoxication (*P* = 0.009). This observation is not surprising because shock is associated with systemic hypoperfusion and progressive failure of multiple organs. Apart from shock as a risk factor, Acikalin et al.^29^ reported that respiratory failure necessitating mechanical ventilatory support, comorbidities, a long hospital stay, elevated creatinine, a low Glasgow Coma Scale score and low pseudocholinesterase levels were poor prognostic factors for mortality after organophosphate intoxication. Furthermore, Tang et al.^30^ revealed that high 6-h postadmission blood lactate levels, low blood pH, and low postadmission 6-h lactate clearance rates were poor prognostic factors for mortality. Finally, a previous study from our team^31^ also demonstrated that hypotension, respiratory failure, coma, and corrected QT interval prolongation were significant risk factors for mortality in patients with organophosphate intoxication. Nevertheless, the study was performed on the general population, not the elderly population.

In theory, shock is classified into four types based on the underlying cause: hypovolemic, cardiogenic, obstructive, and distributive shock^32^. Nevertheless, the exact pathophysiological mechanisms of shock in patients with organophosphate poisoning remain uncertain. The mechanisms of shock in this research could possibly be explained by a combination of hypovolemic, cardiogenic and distributive factors. First, hypovolemic shock since gastrointestinal symptoms such as emesis and diarrhea were common after organophosphate ingestion (Table 2). Severe hypovolemia, or excessive and quick losses of volume could lead to hypovolemic shock. Second, cardiogenic shock since cardiotoxicity is an emerging entity after organophosphate poisoning. Following acute exposure to organophosphates, acetylcholinesterase inhibition ensues and parasympathetic over-activity prevails. Ludomirsky et al. presented 3 distinct phases of cardiac toxicity after organophosphate poisoning: a brief period of increased sympathetic activity, a prolonged period of parasympathetic activity, and finally, in which QT prolongation is followed by torsade de pointes ventricular tachycardia and ventricular fibrillation^33^. Georgiadis et al. proposed that the cardiovascular effect of organophosphate be divided into the following subclasses, myocardial dysfunction, electrical, biochemical (oxidative damage), anatomical, cytopathological, histopathological, biochemical (functional implications), coronary artery disease, biochemical (lipidemic profile and other), and blood pressure disorders (hypertension, hypotension). Our past study^34^ also confirmed that cumulative mortality was higher among organophosphate-poisoned patients with prolonged QT intervals than among those with normal QT intervals. Third, distributive shock since cases of low peripheral resistance and high cardiac output were reported after organophosphate poisoning^35^.

Approximately half of the elderly organophosphate patients (50.7%) developed aspiration pneumonia. Published incidence rates of aspiration pneumonia following organophosphate intoxication range from 21 to 43.5%^12,36,37^. Aspiration pneumonia remains one of the common complications following organophosphate intoxication. This could be due to excess mucosal fluid secretion and conscious disturbance after intoxication^38^. This higher figure (50.7%) could be explained by a relatively compromised immune profile in elderly adults and the high rate of respiratory failure. Impaired swallowing, decreased pulmonary and immune profiles, and increased comorbidities in elderly adults could explain the high incidence of aspiration pneumonia^7,39^. Nevertheless, pneumonia was not a significant risk factor for mortality after regression analysis. In fact, there were no significant differences in the incidence rates of aspiration pneumonia between survivors and nonsurvivors (*P* = 1.000). This may be due to close monitoring and adequate antibiotic treatment of aspiration pneumonia during hospitalization.

Compared with previous study in the general population^31^, the incidences of respiratory failure, hypotension, intermediate syndrome, delayed neuropathy and mortality between general and elderly population were 51.7% versus 52.1%, 22.9% versus 39.4%, 8.5% versus 15.5%, 2.5% versus 4.2% and 15.3% versus 19.7%, respectively. Therefore, aside from delayed neuropathy, it seems that the medical complications of organophosphate intoxication were severer in elderly than general population. The observation is not surprising because of poorer health status in elderly than general population. Laboratory studies also revealed that aged animals are more sensitive to cholinergic (muscarinic) agonists, even though the receptors are reduced^19^. For example, Karanth et al.^20^ demonstrated that 18-month-old rats were more sensitive to the acute toxicity of parathion than 3-month-old rats. Lesser acetylcholine accumulation is required to elicit similar cholinergic response in the 18-month-old rats, possibly due to aging-associated changes in muscarinic receptor density. The results suggest that elderly people may be more vulnerable to organophosphates.

Finally, the limitations of this study included its retrospective nature and small patient population. In addition, laboratory measurements of individual organophosphate compound were not available in this hospital. Therefore, it was difficult to correlate clinical outcome with the dose of adsorbed pesticide. Further research is warranted.

## Conclusions

The mortality rate for elderly patients with organophosphate intoxication was 19.7%.

Acute cholinergic crisis developed in all cases (100.0%). The complications included respiratory failure (52.1%), aspiration pneumonia (50.7%), acute kidney injury (43.7%), severe consciousness disturbance (25.4%), shock (14.1%) and seizures (4.2%). Some patients also developed intermediate syndrome (15.5%) and delayed neuropathy (4.2%). The analytical results revealed that shock carries a significant risk for mortality. Therefore, prompt diagnosis of organophosphate intoxication, volume replacement for fluid losses followed by inotropic drug infusion such as dopamine as well as administration of antidotes such as anti-cholinergic and oxime drugs, could rapidly alter the course of the disease and prevent the development of fatal complications.

## Methods

### Patients

A total of 71 elderly patients (age ≥ 60 years) with organophosphate poisoning were seen at Chang Gung Memorial Hospital between 2008 and 2017. A cutoff point of 60 years was selected for this study since it is the cutoff value defined by the United Nations^40^. The following data for each patient were traced: age, gender, type of organophosphate, exposure method, job, marital status, underlying systemic disease, smoking habits, alcohol consumption, time elapsed between pesticide ingestion and hospital arrival, clinical manifestations, serum cholinesterase level, hemogram, biochemistry, arterial blood gas, detoxification protocol, hospitalization duration and mortality. Most of the exposures were oral (88.7%) and the remaining was dermal (11.3%). The attribution of exposure routes was derived from clinical history. None of the patients with oral exposure had coingestion. Notably, the quantities of ingested pesticides were imprecise and subjected to recall bias. Some patients ingested organophosphate directly, whereas many patients chose to ingest beverages that were mixed with organophosphates. The laboratory data were collected at admission.

### Inclusion and exclusion criteria

All elderly patients diagnosed with organophosphate intoxication were included for analysis. Patients were excluded from this study if they aged younger than 60 years.

### Diagnosis of organophosphate intoxication

The diagnosis was based on the history of exposure, clinical findings and laboratory parameters. The type of pesticide involved was determined by history, bottle label, or product information provided by patients or family members. Serum cholinesterase activity was analyzed by using an enzymatic method (DF51, Siemens Healthcare Diagnostics, Newark, Delaware, USA). The normal reference values were 7–19 U/mL, and the limit of quantification was 0.8 U/mL^41^.

### Clinical management

Treatments involved gastric lavage with 0.9% normal saline, followed by 1 g/kg activated charcoal and 250 mL magnesium citrate infusion via nasogastric tube. Magnesium citrate was given to prevent constipation after charcoal management. Patients were also treated with antidotes that included anti-cholinergic and oxime drugs. Intravenous atropine was given with a starting dose of 2 mg every 1–2 h, and the dose was titrated to the resolution of respiratory secretions and the cessation of bronchoconstriction. Intravenous pralidoxime therapy was also administered, with 1 g given every 4 h to patients with acute cholinergic crisis. Patients with seizure were given anticonvulsive drug. In our hospital, diazepam was the mainstay of treatment for patients presenting with convulsions. Patients with shock were treated with volume replacement for fluid losses, followed by inotropic drug infusion such as dopamine as it can increase systemic arterial pressure by stimulating the myocardium, without compromising renal blood flow and urine output.

### Classification of pesticide toxicity

Pesticide toxicity was categorized as follows, according to World Health Organization recommendations: Ia extremely hazardous, Ib highly hazardous, II moderately hazardous, III slightly hazardous and U unlikely to present acute hazards^42^.

### Statistical analysis

Categorical variables were presented as numbers with percentages in brackets, while continuous variables were presented as the means and standard deviations. All data were examined for normality of distributions and equality of standard deviations before analysis. Chi-square tests or Fisher’s exact tests were performed to compare categorical variables between patient groups, and Student’s t-tests were used for quantitative variables. An initial univariate Cox regression analysis was applied to compare possible predictive factors of mortality, followed by a multivariate Cox regression to control for possible confounding factors that were significant (*P* < 0.05) in the former model and that met the assumptions of a proportional hazards model. Patients with or without shock were estimated for survival curves with the Kaplan–Meier method and tested with the log-rank test. The results rejecting the null hypothesis with 95% confidence were regarded as significant. The data were tabulated in Microsoft Excel, and all analyses were performed with SPSS 18.0 for Windows (SPSS Inc., Chicago, Illinois, USA).

### Ethical statement

This observational study adhered to the Declaration of Helsinki and was approved by the Medical Ethics Committee of Chang Gung Memorial Hospital, Taiwan. Since this study was a retrospective review of existing medical records, Institutional Review Board approval was acquired but without specific informed consent from the patients. The Institutional Review Board of Chang Gung Memorial Hospital specifically waived the need for consent. The Institutional Review Board number allocated to the study was 201800245B0.
